# Interpregnancy Weight Change and Adverse Birth Outcomes: Cohort Study Using Brazil's Routine Register‐Based Linked Data

**DOI:** 10.1111/mcn.70052

**Published:** 2025-05-28

**Authors:** Aline S. Rocha, Thais Rangel Bousquet Carrilho, Priscila R. F. Costa, Enny S. Paixao, Natanael J. Silva, Helena B. M. da Silva, Ila R. Falcão, Rosemeire L. Fiaccone, Mauricio L. Barreto, Rita de Cássia Ribeiro‐Silva

**Affiliations:** ^1^ Center of Data and Knowledge Integration for Health (CIDACS), Oswaldo Cruz Foundation Salvador Brazil; ^2^ School of Nutrition Federal University of Bahia (UFBA) Salvador Brazil; ^3^ Department of Obstetrics and Gynaecology Faculty of Medicine University of British Columbia Vancouver British Columbia Canada; ^4^ Faculty of Epidemiology and Population Health, London School of Hygiene and Tropical Medicine London UK; ^5^ ISGlobal, Hospital Clínic Universitat de Barcelona Barcelona Spain; ^6^ Department of Statistics Federal University of Bahia (UFBA) Salvador Brazil; ^7^ Institute of Collective Health Federal University of Bahia (ISC/UFBA) Salvador Brazil

**Keywords:** body mass index, low birth weight, macrosomia, pregnancy, preterm birth

## Abstract

The effects of interpregnancy weight change (IPWC) on the risk of adverse birth outcomes in subsequent pregnancies are still not fully understood. Existing studies present conflicting results regarding the association between IPWC and preterm birth, while evidence of its relationship with low birth weight (LBW) or macrosomia is limited, particularly in low‐ and middle‐income countries. This population‐based longitudinal study used Brazil's routine register‐based linked data from 2008 to 2015 to evaluate the association between IPWC and adverse birth outcomes in a subsequent pregnancy. Preterm birth, LBW, and macrosomia were compared across categories of IPWC between pregnancies (including changes in BMI unit, changes in BMI category, and percentage of weight change). Logistic and multinomial logistic regressions were used to estimate the association between IPWC and adverse birth outcomes. We analysed 15,570 live births from 7785 multiparous women. Women who reduced their BMI between pregnancies had an increased chance of delivering preterm neonates (OR 1.27; 95% CI 1.01–1.60) and those who increased their BMI by ≥ 4 units between pregnancies had an increased chance of macrosomia (OR 1.60; 95% CI 1.21–2.12) compared to those who maintained their BMI. Similar results were observed when IPWC was defined as changes in BMI categories and percentage changes in weight. The results of this study show that IPCW were associated with changes in both the newborn's maturity and size in a subsequent pregnancy. These findings support the need to develop experimental studies on the effects of maternal weight management within and between pregnancies to improve outcomes for both mothers and babies.

## Summary

- Pre‐pregnancy body mass index (BMI) deviations are associated with a series of adverse outcomes for both mothers and children. However, the influence of weight change between pregnancies on the risks of preterm birth, low birth weight, or macrosomia in a subsequent pregnancy remains poorly understood, especially in low‐ and middle‐income countries.
- Infants born to women who lost weight between pregnancies had higher chance of preterm birth and low birth weight. Additionally, high weight gain between pregnancies was associated with an increased chance of macrosomia.
- Our findings can bolster the significance of professional guidance regarding weight management between pregnancies, optimising outcomes for both mothers and their infants.
- Future research might explore the impact of interventions aimed at managing weight gain during and between pregnancies to mitigate the risks of preterm birth, low birth weight, and macrosomia.

## 1 Introduction

Adverse birth outcomes, such as preterm birth, low birth weight (LBW), and macrosomia, are considered major health problems with short‐ and long‐term consequences for the children (Blencowe et al. 2019; Koyanagi et al. 2013; Ohuma et al. 2023). In Latin America and the Caribbean, 8.9% of live births were born prematurely in 2020 (Ohuma et al. 2023), and 8.7% were born with LBW in 2015 (Blencowe et al. 2019). The prevalence of macrosomia varies between low‐ and middle‐income countries (LMICs), ranging from 0.5% in India to 14.9% in Algeria (Koyanagi et al. 2013). In Brazil, a population‐based study found that the prevalence of preterm birth and LBW was 9.4% and 9.6%, respectively (Paixao et al. 2021). Additionally, macrosomia's prevalence ranges from 1.7% to 17.8% across various studies in the country (Czarnobay et al. 2019).

Although it is widely understood that pre‐pregnancy overweight or obesity are risk factors for adverse perinatal complications (Liu et al. 2016; Poston et al. 2016), the evidence on the effects of interpregnancy weight change (IPCW)—defined as the difference in weight between consecutive pregnancies (Cnattingius and Villamor 2016; Villamor and Cnattingius 2016)—on the risk of adverse birth outcomes in subsequent pregnancies are still not fully understood, despite growing evidence (Martínez‐Hortelano et al. 2024; Oteng‐Ntim et al. 2018; Teulings et al. 2019; Timmermans et al. 2020). Previous studies offer contradictory results regarding the association between IPWC and preterm birth (Benjamin et al. 2019; Grove et al. 2019; Villamor and Cnattingius 2016; Wallace et al. 2014) and provide limited evidence on the association with LBW (Bogaerts et al. 2013; Ku et al. 2023) or macrosomia (Ku et al. 2023; McBain et al. 2016; McClurg et al. 2022). Moreover, there remains a scarcity of studies examining the direction and magnitude of the association between IPWC among successive pregnancies and its potential impacts on maternal and child health in LMICs. Furthermore, comparisons between studies are hindered by differences in the populations, settings, statistical methods and definitions of IPWC.

Studies assessing IPWC have used varying definitions. For example, some studies define IPWC by units of increase or decrease in BMI (Benjamin et al. 2019; Grove et al. 2019; McBain et al. 2016; Villamor and Cnattingius 2016); others by BMI category changes according to the World Health Organization (WHO) classification (Getahun et al. 2007; Riley et al. 2016), or by percentage weight change (Pole and Dodds 1999; Riley et al. 2016). These metrics capture distinct dimensions of weight change (absolute, categorical, or proportional), which can differentially affect maternal and neonatal outcomes.

Thus, we aimed to evaluate the association between IPWC using different metrics (changes in BMI units, changes in BMI category, and percentage weight change) and adverse birth outcomes (preterm birth, LBW, and macrosomia) in the subsequent pregnancy using administrative‐linked data from Brazil. Considering that this is the first study to assess IPWC and adverse birth outcomes within a poor population from a middle‐income country with pronounced social and health disparities (Rebouças et al. 2022), these findings will be valuable in identifying categories of women who would benefit most from public health interventions or advice within and between pregnancies to improve childbirth and health outcomes in LMICs.

## 2 Methods

### 2.1 Study Design and Population

This population‐based longitudinal study was conducted using linked data from three different Brazilian databases: the 100 million Brazilian Cohort baseline, the National System of Live Births in Brazil (SINASC, Sistema de Informação sobre Nascidos Vivos), and the Food and Nutrition Surveillance System (SISVAN, Sistema de Vigilância Alimentar e Nutricional), covering the period from 1 January 2008 to 31 December 2015.

The 100 Million Brazilian Cohort baseline, built mainly from the Cadastro Único (CadÚnico) registration system (Barreto et al. 2021), covers the Brazilian population who applied for any social benefit from the government (Cidacs 2018). The second administrative dataset used in this study was the SINASC, which captures data derived from the Declaration of Live Birth, a form completed by the healthcare professional present during the delivery (Brazil 2022). The SINASC covers approximately 94% of the Brazilian live births (Szwarcwald et al. 2019). Finally, the third dataset was the SISVAN, designed to record anthropometric (weight and height) and dietary intake data at all stages of life of individuals who use primary public health care services (Brazil 2017).

The linkage process involved two distinct approaches. First, a deterministic linkage was executed between the 100 Million Brazilian Cohort baseline and the SISVAN, as both datasets feature the Social Identification Number (SIN), a unique identifier assigned to each individual. Second, a nondeterministic linkage based on the similarity index was employed (Almeida et al. 2020). For this step, the following variables referring to the mother at delivery were used: full name, date of birth (or age in completed years when the date of birth was missing), and municipality of residence. This method facilitated the linkage of the 100 Million Brazilian Cohort baseline to a subset of individuals from the SISVAN who did not have a SIN, and the linking of the resulting database (100 Million Brazilians Cohort baseline + SISVAN) with the SINASC. The linkage process used CIDACS‐RL (Record Linkage), an in‐house record linkage tool developed to link large‐scale administrative datasets at CIDACS (Barbosa et al. 2020). A total of 257,049,913 records were linked, representing 83.66% of the dataset. This includes the sum of linked records from both linkage approaches and all follow‐up records from SISVAN, encompassing data on children, adults, and elderly individuals. Using the optimal cut‐off point (0.941), the linkage accuracy was high, with a specificity of 93.80% and a sensitivity of 97.20%. The Receiver Operating Characteristic (ROC) curve illustrating these metrics is presented in Supporting Information: Figure 1.

**Figure 1:**
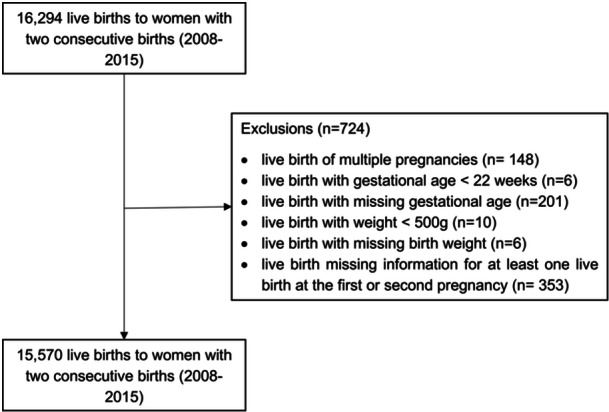
Study population flow diagram, 2008–2015.

In this study, we identified successive pregnancies using the unique maternal identifier and the newborn's date of birth. We included women aged 10–49 years who had their first two consecutive live births from 2008 to 2015 and provided complete information on pre‐pregnancy height and weight for both pregnancies. We excluded all individuals with multiple births, as this condition is strongly associated with an increased risk of adverse birth outcomes (Getachew et al. 2024; Whittaker et al. 2023); missing data on birth weight and gestational age; records with birth weight < 500 g and gestational age at birth < 22 weeks, considering survival and biological implausibility limits (Patel et al. 2017; Upadhyay et al. 2015); and missing information for at least one live birth in the first or second pregnancy in the period (Figure 1). This study adhered to the Reporting of Studies Conducted using Observational Routinely‐collected Data (RECORD) statement.

### 2.2 Main Variables

Pre‐pregnancy BMI was calculated by dividing the weight in kilograms (kg) by height in metres squared (m^2^), with both weight and height data obtained from the SISVAN records. Pre‐pregnancy weight was self‐reported for 40.8% of individuals in their first pregnancy and 58.2% in their second pregnancy. When pre‐pregnancy weight was unavailable, the first weight measurement taken up to the eighth week of gestation was used (59.2% in the first pregnancy and 41.8% in the second pregnancy) (Rangel Bousquet Carrilho et al. 2020).

For adult women, pre‐pregnancy BMI was categorised according to the WHO cutoffs as underweight (< 18.5 kg/m^2^), normal weight (18.5–25 kg/m^2^), overweight (25–30 kg/m^2^), and obesity (≥ 30 kg/m^2^) (WHO 1995). For adolescents, pre‐pregnancy BMI was classified using the WHO BMI‐for‐age curves for girls: underweight (z‐score < −2), normal weight (z‐score ≥ −2 and ≤ 1), overweight (z‐score > 1 and ≤ 2), and obesity (z‐score > 2) (WHO 2007).

IPWC was measured using three different metrics to assess different aspects of weight change between pregnancies:

1. Change in BMI units: calculated as the difference between BMI in the second and first pregnancies during the cohort period. The changes were categorised as follows: loss of BMI of more than 2 units (< −2); loss of BMI from 2 to less than 1 unit (−2 to < −1); loss of 1 BMI unit to gain of less than 1 BMI unit (−1 to < 1); gain of 1 to less than 2 BMI units (1 to < 2); gain of 2 to less than 4 BMI units (2 to < 4); and, gain of 4 or more BMI units (≥ 4). The reference category for the analyses was the range from −1 to less than 1 BMI unit of change (−1 to < 1 kg/m^2^) (Benjamin et al. 2019; Grove et al. 2019; McBain et al. 2016; Villamor and Cnattingius 2016). This metric captures absolute changes in BMI between pregnancies, allowing for a detailed analysis of the effects of different magnitudes of weight change on adverse birth outcomes.
2. Changes in BMI category (WHO classification): created based on changes in the nutritional status of women between two consecutive pregnancies, according to the WHO BMI classification. The categories were: underweight to underweight; underweight to normal weight; underweight to overweight; underweight to obesity; normal weight to underweight; normal weight to normal weight; normal weight to overweight; normal weight to obesity; overweight to underweight; overweight to normal weight; overweight to overweight; overweight to obesity; obesity to underweight; obesity to normal weight; obesity to overweight; obesity to obesity. Women with normal BMI in both pregnancies were considered the reference category in the analyses. This metric captures changes in women's nutritional status between two pregnancies. This approach allows us to understand how the transition between categories, such as from normal weight to overweight or from obesity to normal weight, may be associated with different neonatal risks.
3. Percentage weight changes between pregnancies: calculated as the difference between pre‐pregnancy weight in the second and first pregnancies, expressed as a percentage. The categories were defined as: weight loss, gained 0%–8.62%, or gained > 8.62%. The median percentage of weight gain was calculated among all women who gained weight between the second and first pregnancies (Riley et al. 2016). This metric represents a relative measure of weight change within our study sample, allowing us to understand how different levels of weight gain or loss may influence adverse birth outcomes.

The adverse birth outcomes in the subsequent pregnancy adopted in this study included preterm birth, defined as gestational age < 37 completed weeks (WHO 1977); low birth weight (LBW), defined as birth weight < 2500 g (Koyanagi et al. 2013); and macrosomia, defined as birth weight ≥ 4000 g (Koyanagi et al. 2013).

### 2.3 Other Variables

The following variables, reported during the first pregnancy, were considered in the characterisation of the study population: region of residence (North, Northeast, Southeast, South, and Center‐West), residence area (urban and rural), household overcrowding (≤ 2 inhabitants per room, > 2 inhabitants per room), maternal education (< 3 years, 4–7 years, ≥ 8 schooling years), marital status (married or in a stable relationship; single, divorced or widowed), maternal race/ethnicity (White, Mixed‐race [“*Parda”*], Black), maternal age at delivery (14–19, 20–34, and 35–49 years old), number of prenatal visits (none, 1–3; 4–7, ≥ 7 visits), type of delivery (vaginal or caesarean), and pre‐pregnancy BMI (underweight, normal, overweight, or obesity). We also considered as covariates maternal race/ethnicity (White, Parda, and Black) and interbirth interval (< 24 months or ≥ 24 months). Details on the creation or recategorization of each variable are provided in Supporting Information: Frame 1.

### 2.4 Statistical Analysis

Maternal and pregnancy characteristics were summarised using absolute (*n*) and relative (%) frequencies and are presented according to the categories of adverse birth outcomes. To test our hypothesis that IPWC, measured by different metrics, is associated with the selected adverse outcomes, we employed logistic or multinomial logistic regressions to estimate odds ratios (OR) and 95% confidence intervals (95% CI). Crude and adjusted ORs were estimated. For the exposure change in BMI category, we excluded some categories from the analyses due to small sample sizes (defined as ≤ 3 events for the outcomes of interest). Based on the published literature, biological plausibility, and the set of time‐dependent variables available in our dataset, the covariates from the first pregnancy included in our model were maternal age at delivery, maternal education, area of residence, maternal race, and interbirth interval. Considering pre‐pregnancy BMI as a potential effect modifier between IPCW and adverse birth outcomes (Riley et al. 2016; Wallace et al. 2014), we examined the association between percentage weight changes between pregnancies and adverse birth outcomes in the subsequent pregnancy, stratified by pre‐pregnancy BMI category in the first pregnancy. Additionally, to account for a potential influence of maternal age on the relationship between weight gain during pregnancy and adverse birth outcomes, we assessed the association between IPCW and adverse birth outcomes stratified by maternal age at the time of the first pregnancy. However, this analysis was only feasible for IPWC as percentage weight change, due to sample size issues.

## 3 Results

Between 2008 and 2015, 16,294 live births were retrieved from women with two consecutive singleton first births. After applying the exclusion criteria, 15,570 live births from 7785 women were included in this study (Figure 1).

Most women resided in the Northeast (34.8%) and Southeast (28.7%) regions of the country and in urban areas (68.8%) in their first pregnancy. They experienced less home overcrowding (< 2 inhabitants per room, 88.9%), were single, widowed, or divorced (65.0%), had more than four schooling years (90.0%), belonged to mixed‐race [“*Parda”*] race/ethnicity group (53.9%), were aged between 20 and 34 years (58.0%), underwent more antenatal visits (64.2%), had a vaginal birth (65.5%), a normal pre‐pregnancy BMI (64.3%), and a birth interval ≥ 24 months (62.7%) (Table 1). In the subsequent pregnancy, 11.6% of the neonates were preterm, 5.1% had LBW, and 6.5% had macrosomia (Table 1). The prevalence of preterm birth, LBW, or macrosomia differed according to the region and area of residence, maternal age, education level, and maternal race/ethnicity (Table 1).

**Table 1:** Maternal and pregnancy characteristics by adverse birth outcomes in the second pregnancy, Brazil, 2008–2015.

First pregnancy variables	Total births	Term	Preterm birth	Normal birth weight	Low birth weight	Macrosomia
*N* (%)	7785 (100)	6882 (88.4)	903 (11.6)	6880 (88.4)	396 (5.1)	507 (6.5)
Residence region						
North	574 (7.4)	496 (7.2)	78 (8.64)	503 (7.3)	31 (7.9)	40 (7.9)
Northeast	2712 (34.8)	2385 (34.7)	327 (36.21)	2389 (34.7)	111 (28.0)	212 (41.8)
Southeast	2232 (28.7)	1.97 (28.6)	262 (29.01)	1972 (28.7)	145 (36.6)	115 (22.7)
South	1943 (25.0)	1748 (25.4)	195 (21.59)	1737 (25.2)	93 (23.5)	113 (22.3)
Center‐West	324 (4.2)	283 (4.1)	41 (4.54)	281 (4.1)	16 (4.0)	27 (5.3)
Missing^1^	42 (0.5)					
Residence area						
Urban	5224 (68.8)	4586 (68.3)	638 (72.25)	4625 (68.9)	282 (72.7)	317 (64.2)
Rural	2.37 (31.2)	2125 (31.7)	245 (27.75)	2087 (31.1)	106 (27.3)	177 (35.8)
Missing^1^	191 (2.5)					
Household overcrowding (inhabitants per room)						
< 2	6596 (88.9)	5845 (89.2)	751 (87.02)	5825 (88.8)	334 (88.8)	437 (90.5)
≥ 2	821 (11.1)	709 (10.8)	112 (12.98)	733 (11.2)	42 (11.2)	46 (9.5)
Missing^1^	368 (4.7)					
Marital status						
Married/Civil partnership	2680 (35.1)	2384 (35.23)	296 (33.33)	2353 (34.8)	142 (36.5)	185 (36.9)
Single/widow/divorced	4975 (65.0)	4383 (64.77)	592 (66.67)	4412 (65.2)	247 (63.5)	316 (63.1)
Missing^1^	130 (1.7)					
Maternal education (years)						
Up to 3	771 (10.1)	665 (9.8)	106 (11.98)	665 (9.8)	57 (14.6)	49 (9.9)
04–07	3208 (40,0)	2839 (42.1)	369 (41.69)	2846 (42.2)	151 (38.7)	211 (42.5)
≥ 8	3656 (47.9)	3246 (48.1)	410 (46.33)	3238 (48.0)	182 (46.7)	236 (47.6)
Missing^1^	150 (1.9)					
Maternal race/ethnicity						
White	3146 (41.8)	2806 (42.1)	340 (39.17)	2812 (42.2)	168 (44.1)	166 (33.9)
Mixed‐race “*Parda*”	4056 (53.8)	3573 (53.6)	483 (55.65)	3555 (53.4)	198 (52.0)	303 (62.0)
Black	330 (4.4)	285 (4.3)	45 (5.18)	295 (4.4)	15 (3.9)	20 (4.1)
Missing^1^	253 (3.3)					
Maternal age (years)						
14–20	2955 (58.0)	4024 (58.5)	490 (54.26)	3962 (57.6)	217 (54.8)	335 (66.1)
20–34	4514 (38.0)	2591 (37.6)	364 (40.31)	2650 (38.5)	156 (39.4)	149 (29.4)
35–49	316 (4.0)	267 (3.9)	49 (5.43)	270 (3.9)	23 (5.8)	23 (4.5)
Missing^1^	0 (0.0)					
Number of prenatal visits						
Up to 3	424 (5.5)	354 (5.2)	70 (7.79)	366 (5.3)	30 (7.6)	28 (5.5)
4–6	2352 (30.4)	2018 (29.5)	334 (37.15)	2043 (29.9)	137 (34.8)	172 (34.0)
7+	4967 (64.1)	4472 (65.3)	495 (55.06)	4434 (64.8)	227 (57.6)	306 (60.5)
Missing^1^	42 (0.5)					
Type of delivery						
Vaginal	5090 (65.5)	4471 (65.0)	619 (68.70)	4515 (65.7)	252 (63.8)	323 (63.7)
Caesarean section	2686 (34.5)	2404 (35.0)	282 (31.30)	2359 (34.3)	143 (36.2)	184 (36.3)
Missing^1^	9 (0.12)					
BMI 1st pregnancy (kg/m^2^)						
< 18.5	376 (4.8)	327 (4.8)	49 (5.43)	332 (4.8)	32 (8.1)	12 (2.4)
18.5 to < 25	5007 (64.3)	4417 (64.2)	590 (65.34)	4473 (65.0)	266 (67.2)	268 (52.9)
25 to < 30	1695 (21.8)	1507 (21.9)	188 (20.82)	1500 (21.8)	63 (15.9)	132 (26.0)
≥ 30	707 (9.1)	631 (9.2)	76 (8.42)	577 (8.4)	35 (8.8)	95 (18.7)
Missing^1^	0 (0.0)					
Interpregnancy interval (months)						
< 24	2763 (35.5)	2390 (34.7)	373 (41.3)	2433 (35.4)	165 (41.7)	165 (32.5)
≥ 24	5002 (64.5)	4492 (65.3)	530 (58.7)	44495 (65.6)	231 (58.3)	342 (67.5)
Missing^1^	0 (0.0)					

*Note:* ^1^Percentage was not included when calculating the categories.

Considering the changes in units of BMI, 20.6% of women lost weight (BMI < −1 unit), 49.1% gained weight (> 1 unit of BMI), and 30.4% of women maintained their weight (loss of 1 BMI unit to gain of less than 1 BMI unit) during pregnancies. Regarding BMI changes based on the WHO classification, 9.9% of women reduced their BMI enough to be moved to a lower category, 22.6% increased their BMI and progressed to a higher category, and 67.5% remained within the same classification of BMI in both pregnancies. For the percentage change in weight between pregnancies, 28.7% of women lost weight, 35.8% gained between 0% and 8.62% of weight, and 35.7% gained more than the median gain (8.62%). Overall, individuals who experienced an increase in BMI or weight between pregnancies had a higher prevalence of macrosomia. In contrast, those who had a reduction in BMI or weight had a higher prevalence of premature birth and LBW (Supporting Information: Table 2).

### 3.1 Change in BMI Units and Adverse Birth Outcomes

**Figure 2:**
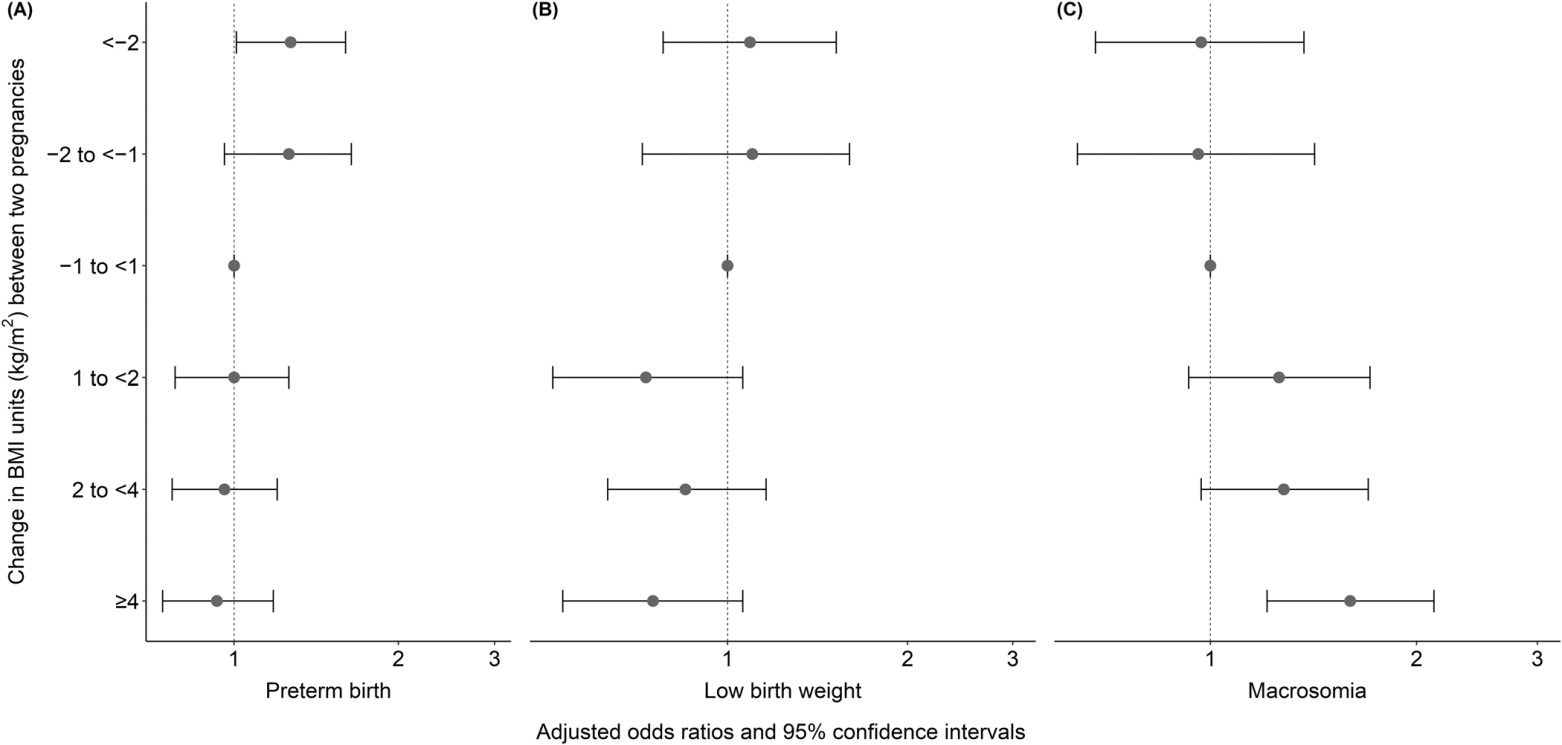
Change in body mass index (BMI) units (kg/m^2^) between pregnancies and birth outcomes in the subsequent pregnancy, Brazil, 2008–2015. (A) Preterm birth; (B) low birth weight; (C) macrosomia. *Note:* Analysis was adjusted for residence area, maternal education, and maternal age at delivery at the first pregnancy, maternal race and interpregnancy interval.

Infants born in the subsequent pregnancies of women who reduced their BMI (< −2 units of BMI) between pregnancies had an increased chance of prematurity (OR 1.27; 95% CI 1.01–1.60) when compared to those born to women with stable BMI (BMI unit change from −1 to < 1 kg/m^2^) (Figure 2A). Conversely, a high interpregnancy BMI increase (≥ 4 units of BMI) was associated with an increased chance of live births with macrosomia in the subsequent pregnancy (OR 1.60 IC 95% 1.21–2.12) compared to those born to women with a stable BMI (BMI change from −1 to < 1 kg/m^2^) (Figure 2C). However, no significant associations were observed between changes in BMI units in the two pregnancies and LBW in the subsequent pregnancy (Figure 2B).

### 3.2 Changes in BMI Category and Adverse Birth Outcomes

**Figure 3:**
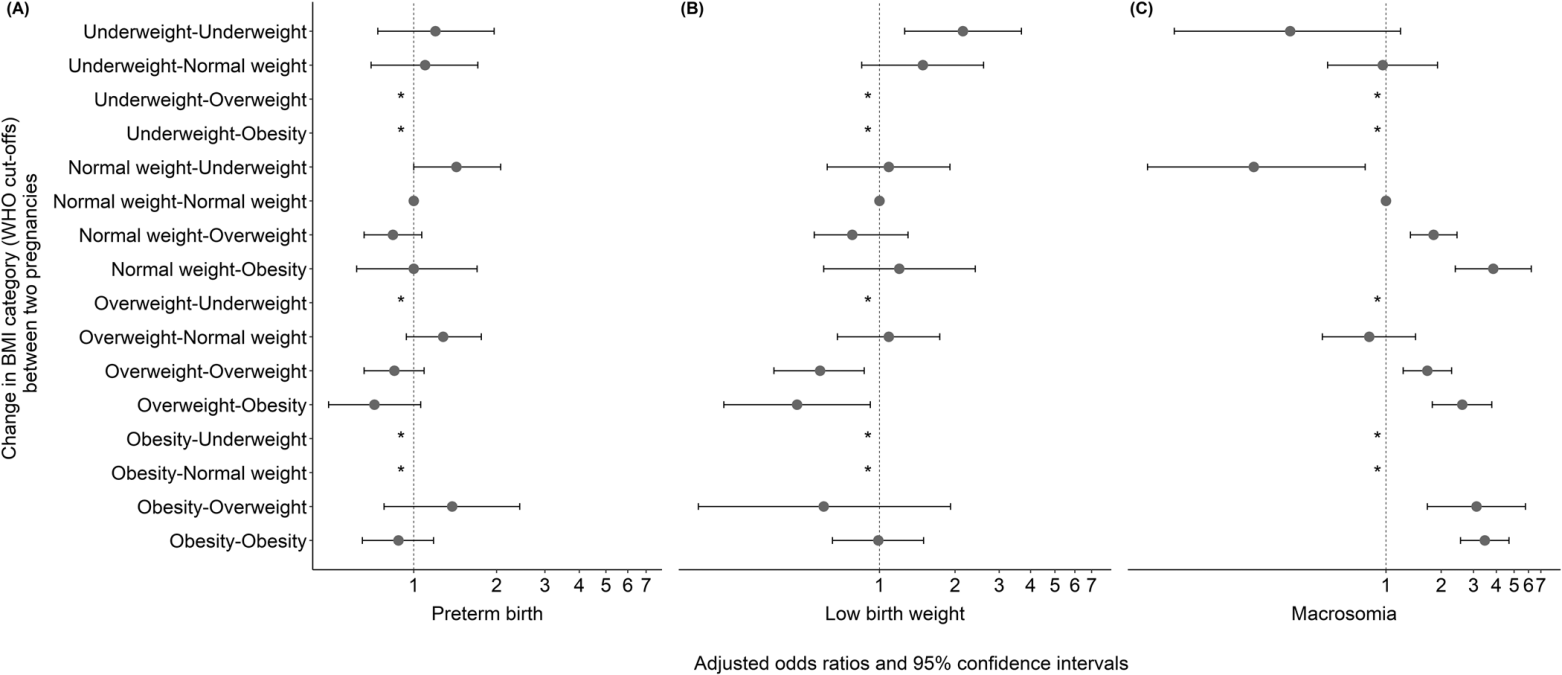
Change in body mass index (BMI) category (WHO) between pregnancies and birth outcomes in the subsequent pregnancy, Brazil, 2008–2015. (A) Preterm birth. (B) Low birth weight. (C) Macrosomia. *Note:* Analysis was adjusted for residence area, maternal education, and maternal age at delivery at the first pregnancy, maternal race and interpregnancy interval. *These categories were excluded from the analyses because of insufficient sample sizes.

A change in BMI category from normal weight to underweight between the first two pregnancies increased the chance of preterm birth in the subsequent pregnancy (OR 1.43; 95% CI 1.00–2.31) compared to women with normal weight during the first and second pregnancies (Figure 3A). Similarly, neonates born to women who were underweight in both first and second pregnancies had higher chances of LBW in the subsequent pregnancy (OR 2.15; 95% CI 1.26–3.68) when compared to those born to women with normal weight in both pregnancies (Figure 3B). Conversely, infants born to women who were overweight in both pregnancies and those who had an increase in BMI from overweight to obesity showed a reduced chance of LBW in the subsequent pregnancy (OR 0.58; 95% CI 0.38–0.87; OR 0.47; 95% CI 0.24–0.97, respectively) when compared to women with normal BMI before both pregnancies (Figure 3B).

Regarding macrosomia, live births from women with a change in BMI category from normal weight to underweight showed a reduced chance of this outcome in the second pregnancy (OR 0.19; 95% CI 0.05–0.77) compared to women with a normal BMI in both pregnancies (Figure 3C). However, the chance of macrosomia was higher among live births from women who changed their BMI category to a higher level [normal weight to overweight (OR 1.89; 95% CI 1.36–2.46); normal weight to obesity (OR 3.85; 95% CI 2.39–6.21); and overweight to obesity (OR 2.61; 95% CI 1.79–3.98)] (Figure 3C).

Additionally, children of women who changed their BMI category from obesity to overweight showed increased chances of macrosomia (OR 3.12; 95% CI 1.68–5.77) (Figure 3C). Neonates born to women with were overweight or obesity in both pregnancies had higher chances of macrosomia in the subsequent pregnancy than those born to women with normal BMI in both pregnancies. Specifically, the odds of macrosomia were 1.68 times higher (95% CI 1.24–2.28) for women who remained overweight and 3.46 times higher (95% CI 2.55–4.69) for those who remained with obesity (Figure 3C).

### 3.3 Percentage Weight Changes Between Pregnancies and Adverse Birth Outcomes

**Figure 4:**
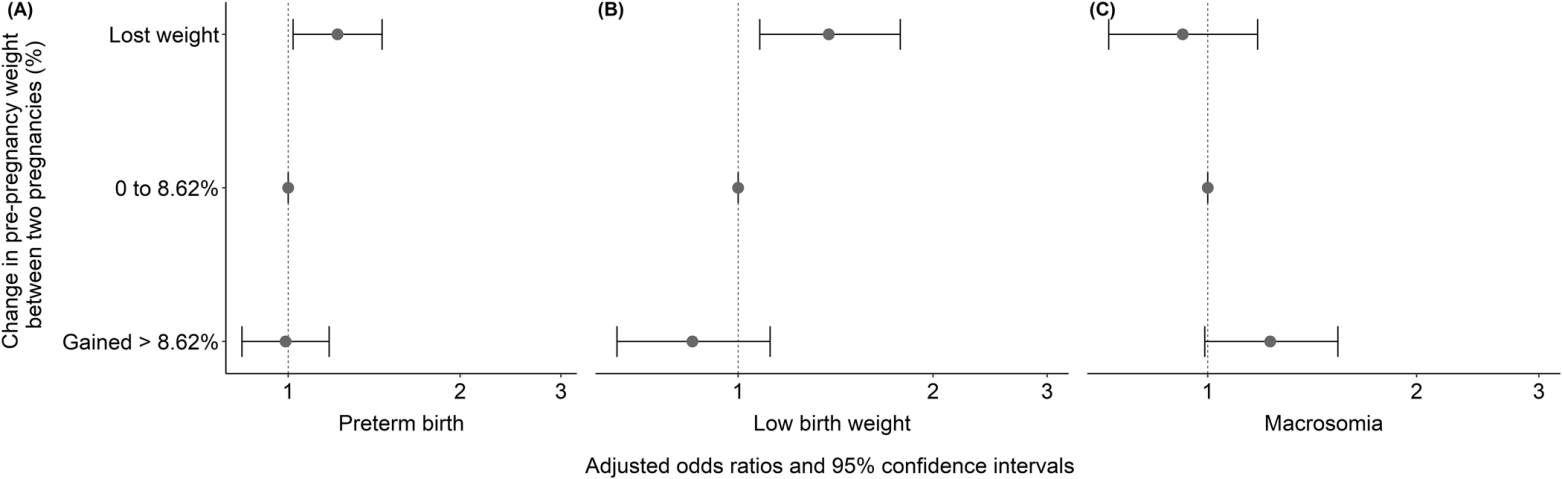
Percentage change weight between pregnancies and birth outcomes in the subsequent pregnancy, Brazil, 2008–2015. (A) Preterm birth. (B) Low birth weight. (C) Macrosomia. *Note:* Analysis was adjusted for residence area, maternal education, and maternal age at delivery at the first pregnancy, maternal race and interpregnancy interval.

Neonates born to women who lost weight between pregnancies had an increased chance of preterm birth (OR 1.22; 95% CI 1.02–1.45) compared to those born to women who gained between 0% and 8.62% (Figure 4A). Similarly, infants born to women who lost weight until the beginning of the subsequent pregnancy had an increased chance of LBW (OR 1.38; 95% CI 1.08–1.78) compared to those born to women who gained between 0% to 8.62% (Figure 4B). No significant associations were observed between the percentage change in weight between the two pregnancies and macrosomia in the subsequent pregnancy (Figure 4C).

### 3.4 Analysis by Pre‐Pregnancy BMI and Maternal Age

When the analysis was stratified by pre‐pregnancy BMI category during the first pregnancy, women with normal weight and who lost weight between pregnancies had a greater chance of having babies with LBW in the subsequent pregnancy (OR 1.44; 95% CI 1.04–1.99) compared to those who gained weight between 0% and 8.62% (Supporting Information: Table 4). However, no associations were identified between the percentage change in weight between pregnancies and preterm birth and macrosomia in the subsequent pregnancy (Supporting Information: Tables 3–5).

For the percentage of weight change, when we stratified the analysis by maternal age at the time of the first pregnancy, we observed that there were no differences between the associations observed for adolescents or adults (Supporting Information: Table 6).

## 4 Discussion

### 4.1 Main Findings

We investigated how IPWC relates to adverse birth outcomes in the subsequent pregnancy in a poor population from a middle‐income country, considering three different metrics for IPWC. The selected metrics, grounded in commonly used methods in the literature, enabled a nuanced exploration of how various patterns of IPCW influence neonatal health. By combining three approaches—change in BMI units, change in BMI category, and percentage weight change—we aimed to understand how each pattern of weight change, whether expressed in absolute, categorical, or proportional terms, impacts adverse birth outcomes.

The results from these different metrics consistently showed that IPCW were associated with preterm birth, LBW, and macrosomia. Infants born to women who experienced weight decrease between pregnancies had higher chances of preterm birth and LBW compared to those born to women who maintained a stable weight. Additionally, weight increase pregnancies was associated with an increased chance of macrosomia. Higher chances of this outcome were also observed among women with overweight and obesity in their first pregnancy who remained in the same BMI category in the subsequent one.

### 4.2 Comparison With Literature

Our findings about decreases in weight between pregnancies and increased odds of preterm birth are consistent with previous studies (Benjamin et al. 2019; Timmermans et al. 2020; Villamor and Cnattingius 2016; Wallace et al. 2014). A meta‐analysis showed that a loss of BMI > 1 kg/m^2^ between pregnancies was associated with a higher risk of preterm birth (OR 1.41; 95% CI 1.06–1.89) (Timmermans et al. 2020). However, in another recent meta‐analysis, interpregnancy weight loss was not associated with PTB risk (OR: 1.06; 95% CI 0.97–1.15) (Martínez‐Hortelano et al. 2024).

We also observed that infants born to women with underweight in the first and second pregnancies, as well as those women who lost weight up to the beginning of the subsequent pregnancy, had a higher chance of LBW in the subsequent pregnancy. This is consistent with the literature available for high‐income countries. Bogaerts et al. (2013) and Ku et al. (2023) showed that women who experienced weight loss between pregnancies (> 1 kg/m^2^) had increased odds (OR 2.22; 95% CI 1.41–3.51) and risks (RR 1.36; 95% CI 1.02–1.80) of LBW, respectively, compared to women with stable BMI.

We also observed that a high increase in BMI between pregnancies (≥ 4 units) was associated with increased chances of macrosomia. Moreover, being classified with overweight or obesity in both pregnancies increased the chances of macrosomia. These results are consistent with previous studies. Martínez‐Hortelano et al. in their recent systematic review with meta‐analysis, observed that interpregnancy weight gain was associated with a higher risk of macrosomia, identifying a summary relative risk of 1.04 (95% CI: 1.01–1.07), a considerably lower estimate than those observed in our study (Martínez‐Hortelano et al. 2024).

In an analysis by pregestational BMI at the beginning of the previous pregnancy, we observed that women with normal weight in the first pregnancy who lost weight between pregnancies had a higher likelihood of having LBW infants in their second pregnancy. This finding is consistent with previous studies (Bogaerts et al. 2013; Ku et al. 2023), although the IPCW method used in those studies differed from ours.

The mechanisms that explain these associations are not fully understood. For preterm birth and LBW, it is hypothesised that micro and macronutrient deficiencies can result in poor placental function, insufficient nutrients for the growing foetus or an increased risk of infection. These factors could serve as intermediaries between maternal malnutrition, reduced gestational age and low weight at birth (Alwan et al. 2020; Neggers and Goldenberg 2003). Additionally, some studies speculate whether the observed associations are true or a reflection of residual confounding from unmeasured factors, such as psychosocial stress and poor mental and physical health, which could contribute to both weight loss and preterm birth (Grove et al. 2019; Rocha et al. 2022). For macrosomia, Wallace et al. (Wallace et al. 2014) suggested that placental weight could be an important mediator of the relationship between interpregnancy weight gain and increased birth weight, which could help to explain the association between IPWC and macrosomia. Also, weight loss in women with a normal BMI in the first pregnancy may indicate a reduction in maternal energy reserves, potentially compromising the availability of essential nutrients for foetal growth in the subsequent pregnancy (Neggers and Goldenberg 2003).

### 4.3 Limitations and Strengths

In this study, some limitations are noted. First, administrative data are typically not collected for research purposes and may be subject to limitations related to missing data, potential underestimation, and classification errors, particularly concerning adverse birth outcomes. These errors are likely to be non‐differential and unlikely to introduce bias in the measurement of association, though the absolute measures of risk/odds might be underestimated. Additionally, some variables used in our study contained missing data, which were addressed by applying a complete case analysis.

Another limitation of our study is the absence of potential confounders, as our dataset did not include information on maternal health conditions (e.g., diabetes or infections), access to healthcare services, or specialised care during high‐risk pregnancies. We also lacked data on interventions aimed at body weight control during or after the first pregnancy or information on cases of postpartum weight retention, which could influence our results. However, a recent study on the association between weight gain and adverse neonatal outcomes has shown that adjusting regression models for behavioural, psychosocial, and environmental measurements had a minimal impact on the estimated associations (Bodnar and Hutcheon 2023). Thus, it is reasonable to believe that the lack of the aforementioned confounding variables had a minimal impact on our findings as well.

Furthermore, it is important to consider that factors contributing to weight gain or loss during pregnancy may directly influence adverse birth outcomes, rather than the weight change itself (Tennant et al. 2023; Tennant et al. 2022). This underscores the need for caution when interpreting these associations, as analyses of change scores in observational studies do not directly estimate causal effects.

We were also unable to evaluate additional adverse birth outcomes, such as small or large for gestational age. Specifically, until 2010, gestational age at birth was collected at wide intervals of gestational weeks in SINASC, which limited our ability to assess these outcomes. Furthermore, we were unable to classify preterm birth subtypes (spontaneous or medically indicated) due to the absence of detailed information in our dataset. This lack of granularity constrained our ability to conduct more nuanced analyses of specific outcomes.

Maternal pre‐pregnancy weight was mostly based on self‐reported information, which is subject to recall bias that may lead to incorrect classifications of maternal BMI categories. However, the results of a previous study using SISVAN data revealed that self‐reported pre‐pregnancy weight is in good agreement with weight measured in the first trimester (Carrilho et al. 2022). Furthermore, the small number of women in certain subgroups limited our ability to obtain precise association estimates, resulting in wide confidence intervals. We excluded these categories from our analyses, which restricted a more detailed examination of BMI category changes. However, the low number of individuals in certain BMI change categories, such as underweight to overweight, or underweight to obesity, is expected. This phenomenon arises because significant weight changes are required for an individual to shift their BMI classification by two or three categories. For example, if we consider a woman who is 170 cm tall, weighs 45 kg, and has a BMI of 15.6 kg/m^2^ (underweight) in the first pregnancy, she will need to gain 30 kg between pregnancies to move into the overweight category.

Finally, this study was carried out among the poorest population of an upper middle‐income country with a history of great social and health inequalities (Rebouças et al. 2022). Therefore, our findings are likely to be most applicable to populations with similar socioeconomic conditions.

Despite these limitations, the use of combined administrative data offers certain advantages, such as a large sample, which enabled us to explore the effects of three distinct metrics to capture various dimensions of IPWC and to evaluate their association with adverse birth outcomes. These findings underscore the potential of administrative datasets as a valuable resource in population health research. We provided valuable insights into the effects of interpregnancy weight changes on adverse birth outcomes, which might not have been feasible with smaller, expensive, prospectively collected cohorts.

Additionally, by using these three approaches, we offer a comprehensive analysis of how varying patterns of weight change influence adverse birth outcomes. This depth of analysis adds robustness to the existing literature, particularly in a low‐income population setting. The change in BMI units provides a quantitative measure of variation; the categorical BMI shifts capture clinically relevant transitions between BMI classes; and the percentage weight change highlights relative variations in maternal weight.

## 5 Conclusion

Neonates born to women who experienced a decrease in BMI or weight loss between pregnancies had higher chances of preterm birth and LBW in the subsequent pregnancy compared to those born to women with stable weight between pregnancies. Additionally, high interpregnancy weight gain also increased the chances of macrosomia in neonates born in the subsequent pregnancy, when compared to those born to mothers with a stable weight. Furthermore, being classified with overweight or obesity in both pregnancies increased the odds of macrosomia in a subsequent pregnancy.

These findings support the need for experimental studies that evaluate the effects of maternal weight control within and between pregnancies to improve outcomes for mothers and infants. Our findings serve as a warning of the potential consequences of large weight gains or losses between pregnancies on infant health and reinforce the importance of considering the interpregnancy interval as a critical window for optimising maternal and infant health.

## Author Contributions

Aline S. Rocha and Rita de Cássia Ribeiro‐Silva conceptualised and designed the study, drafted and revised the manuscript. Aline S. Rocha, Thais Rangel Bousquet Carrilho, and Priscila R. F. Costa contributed to statistical analysis. Thais Rangel Bousquet Carrilho, Priscila R. F. Costa, Enny S. Paixao, Natanael J. Silva, Helena B. M. da Silva, Rosemeire L. Fiaccone, and Ila R. Falcão contributed to data interpretation and critically reviewed the intellectual content of the manuscript. Rita de Cássia Ribeiro‐Silva and Mauricio L. Barreto acquired data, contributed to data interpretation, and critically reviewed the intellectual content of the manuscript. All of the authors approved the final, submitted version of this manuscript and accepted accountability for all aspects of this study.

## Ethics Statement

This study was approved by the Research Ethics Committee from the Institute of Collective Health, Federal University of Bahia (ISC‐UFBA) (reference numbers 41695415.0.0000.5030 and 18022319.4.0000.5030) and the School of Nutrition, Federal University of Bahia (ENUFBA) (reference number 67205423.6.0000.5023). This study was waived from obtaining informed consent from participants because it uses only de‐identified electronic data.

## Conflicts of Interest

We declare that Thais Rangel Bousquet Carrilho, coauthor of this manuscript, is an Early Career Researcher Editorial Board Member of *Maternal & Child Nutrition*.

## Supporting information


**Figure S1.** ROC curve of linkage between the 100 million cohort (POP100 V2) and SISVAN‐Antro (Approach 2). Source: Prepared by the CIDACS Data Production Center.
**Frame S1**: Dataset description.
**Table S1:** Maternal and pregnancy characteristics by adverse birth outcomes in the subsequent pregnancy, Brazil, 2008–2015.
**Table S2**: Interpregnancy weight change and adverse births outcomes in the subsequent pregnancy, Brazil, 2008‐2015.
**Table S3**: Interpregnancy weight change and preterm birth in the subsequent pregnancy by pre‐pregnancy BMI category in the 1^st^ pregnancy, Brazil, 2008‐2015.
**Table S4**: Interpregnancy weight change and low birth weight in the subsequent pregnancy by pre‐pregnancy BMI category in the 1^st^ pregnancy, Brazil, 2008‐2015.
**Table S5**: Interpregnancy weight change and macrosomia in the subsequent pregnancy by pre‐pregnancy BMI category in the 1^st^ pregnancy, Brazil, 2008‐2015.
**Table S6**: Interpregnancy weight change and birth outcomes in the subsequent pregnancy by maternal age at in the 1^st^ pregnancy, Brazil, 2008‐2015.

## Data Availability

The data that support the findings of this study are available on request from the corresponding author. The data are not publicly available due to privacy or ethical restrictions. All data supporting this study were obtained from the Center for Data and Knowledge Integration for Health (CIDACS). These were licensed for exclusive use in the present study and, due to the privacy rules of the Brazilian Laws and Ethics Committee, are not openly available. Upon request with adequate justification and approval of an ethics committee, controlled access to data is considered and, if possible, allowed access. The data described in the manuscript, code book and analytical code will be made available upon request to the corresponding author, E‐mail: aline.srocha@fiocruz.br. In order to access the data, each researcher should present a research project, ethical approval, and a data plan to extract an unidentified/anonymized data set for analysis. Further information can be obtained at https://cidacs.bahia.fiocruz.br/acesso-aos-dados/.
