# Supplementary material for: Interpregnancy Weight Change and Adverse Birth Outcomes: Cohort Study Using Brazil's Routine Register‐Based Linked Data
Source: Matern Child Nutr. 2025 May 28;21(4):e70052. doi: 10.1111/mcn.70052 (PMC12454203; doi:10.1111/mcn.70052)
Supplement: Supplementary file 1 — Figure S1. ROC curve of linkage between the 100 million cohort (POP100 V2) and SISVAN‐Antro (Approach 2). Source: Prepared by the CIDACS Data Production Center. Frame S1: Dataset description. Table S1: Maternal and pregnancy characteristics by adverse birth outcomes in the subsequent pregnancy, Brazil, 2008–2015. Table S2: Interpregnancy weight change and adverse births outcomes in the subsequent pregnancy, Brazil, 2008‐2015. Table S3: Interpregnancy weight change and preterm birth in the subsequent pregnancy by pre‐pregnancy BMI category in the 1st pregnancy, Brazil, 2008‐2015. Table S4: Interpregnancy weight change and low birth weight in the subsequent pregnancy by pre‐pregnancy BMI category in the 1st pregnancy, Brazil, 2008‐2015. Table S5: Interpregnancy weight change and macrosomia in the subsequent pregnancy by pre‐pregnancy BMI category in the 1st pregnancy, Brazil, 2008‐2015. Table S6: Interpregnancy weight change and birth outcomes in the subsequent pregnancy by maternal age at in the 1st pregnancy, Brazil, 2008‐2015. [file MCN-21-e70052-s001.docx]

**Supplementary material**

**Title:** Interpregnancy weight change and adverse birth outcomes: cohort study using Brazil's routine register-based linked data


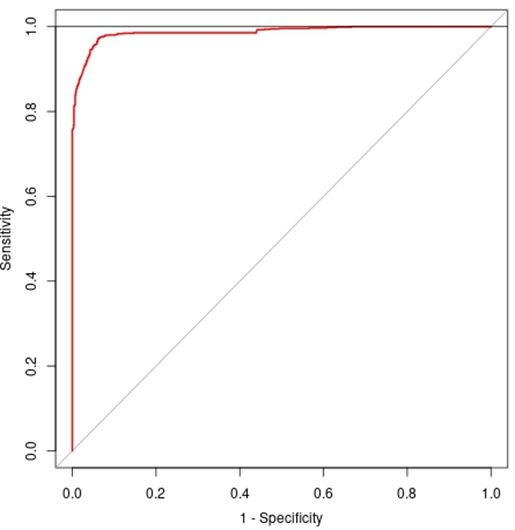


**Figure S1.** ROC curve of linkage between the 100 million cohort (POP100 V2) and SISVAN-Antro (Approach 2).

Source: Prepared by the CIDACS Data Production Center.

**Frame S1**: Dataset description

| **Data source** | **Variables in the dataset/categories** | **Variables used in the study/categories** | **Description** |
| --- | --- | --- | --- |
| **100 million Brazilian Cohort baseline** | Residence area  Urban  Rural | Residence area  Urban  Rural | Used original category |
|  | Number of individuals living in the house | Household overcrowding  ≤2 inhabitants per room  >2 inhabitants per room | Household overcrowding was calculated by dividing the number of people living in the household by the number of rooms. |
|  | Number of rooms |  |  |
| **SISVAN (Sistema de Vigilância Alimentar e Nutricional/Food and Nutrition Surveillance System)** | Weight (kg) | BMI 1st pregnancy (kg/m2)  <18.5  18.5 to <25  25 to <30  ≥30 | It was calculated by dividing the weight in kilograms (kg) by height in meters squared (m^2^). |
|  | Height (cm) |  |  |
|  |  | BMI change (kg/m2)  <-2  ≥-2 to <-1  ≥-1 to <1  ≥1 to /<2  ≥2 to <4  ≥4 | It was calculated as the difference between BMI in the second and first pregnancies during the cohort period. |
|  |  | BMI category change (WHO)  Underweight-Underweight  Underweight-Normal weight  Underweight-Overweight  Underweight-Obesity  Normal weight-Underweight  Normal weight-Normal weight  Normal weight-Overweight  Normal weight-Obesity  Overweight-Underweight  Overweight-Normal weight  Overweight-Overweight  Overweight-Obesity  Obesity-Underweight  Obesity-Normal weight  Obesity-Overweight  Obesity-Obesity | It was created based on changes in the nutritional status of women between two consecutive pregnancies, according to the World Health Organization's BMI classification |
|  |  | % change weight between pregnancies  Lost weight  0 to 8,62%  Gained >8,62% | It was defined as the percentage difference between pre-pregnancy weight in the second and first pregnancies. |
|  | IBGE code of the municipality of residence | Residence region  North,  Northeast, Southeast,  South,  Center-West | It was created from the IBGE code of the mother's municipality of residence. To do this, the first two digits of the IBGE code, which identify the state, were extracted, and each state was associated with its respective geographic region of Brazil. |
| **SINASC (Sistema de Informação sobre Nascidos Vivos/Information System of Live Birth)** | Marital status  Single  Married  Widowed  Legally separated/divorced  Stable union | Marital status  Married/ Civil partnership  Single/widow/divorced | It was reclassified into two main categories |
|  | Maternal race/ ethnicity  Black  Parda (“Mixed-race”)  Indigenous  Yellow (“Asian descent”)  White | Maternal race/ ethnicity  White  Mixed-race “Parda”  Black | It was reclassified into three main categories. In this study, we excluded Asians and Indigenous people due to the limited individual size in these categories. |
|  | Maternal education  1 to 3 years  4 to 7 years  8 to 11 years  12 and over | Maternal education (years)  Up to 3  04 to 07  ≥ 8 | It was reclassified into 3 categories |
|  | Gestational age  Less than 22 weeks  22 to 27 weeks  28 to 31 weeks  32 to 36 weeks  37 to 41 weeks  42 weeks and more | Preterm birth  < 37 weeks  37 to 42 weeks | It was created from the variable gestational week and grouped into 2 categories |
|  | Birth weight (g) | Low birth weight  < 2500g  ≥2500 to < 4000 | It was created through birth weight (g) and classified into two categories. |
|  |  | Macrosomia  ≥2500 to < 4000  ≥ 4000g | It was created through birth weight (g) and classified into two categories. |
|  | Mother's date of birth | Maternal age (years)  14 to 20  20 to 34  35 to 49 | Was calculated in years by subtracting the mother’s date of birth from the child’s date of birth |
|  | Child's Date of Birth |  |  |
|  |  | Interpregnancy  interval (months)  <24  ≥24 | It was estimated, in months, by the difference between the birth date of the child in the subsequent pregnancy and the birth date of the child in the previous pregnancy. |
|  | Number of prenatal visits  None  1 to 3  4 to 6  7 and more | Number of prenatal visits  Up to 3  4 to 6  7+ | It was reclassified into 3 categories |
|  | Type of delivery  Vaginal  Cesarean section | Type of delivery  Vaginal  Cesarean section | Used original category |
| IBGE - Instituto Brasileiro de Geografia e Estatística (Brazilian Institute of Geography and Statistics); g – grams, Kg – Kilogram; cm – centimeter; m^2^ - meters squared | | | |

| Table S1: Interpregnancy weight change and adverse births outcomes at the second pregnancy, Brazil, 2008–2015. | | | | | | |
| --- | --- | --- | --- | --- | --- | --- |
|  | **Total births** | **Term** | **Preterm** | **Normal  birth weight** | **Low**  **birth weight** | **Macrosomia** |
| N (%) | 7,785 (100) | 6,882 (88.4) | 903 (11.6) | 6,880 (88,4) | 396 (5.1) | 507 (6.5) |
| BMI change (kg/m2) |  |  |  |  |  |  |
| <-2 | 936,000 (12.0) | 802 (11.7) | 266 (29.5) | 827 (12.0) | 58 (14.7) | 51 (10.1) |
| ≥-2 to <-1 | 663,000 (8.5) | 568 (8.2) | 134 (14.8) | 586 (8.5) | 42 (10.6) | 35 (6.9) |
| ≥-1 to <1 | 2,367 (30.4) | 2,101 (30.5) | 266 (29.5) | 2,100 (30.5) | 136 (34.3) | 131 (25.8) |
| ≥1 to <2 | 1,050 (13.5) | 933 (13.6) | 117 (13.0) | 935 (13.6) | 43 (10.9) | 72 (14.2) |
| ≥2 to <4 | 1,494 (19.2) | 1,339 (19.5) | 155 (17.2) | 1,321 (19.2) | 67 (16.9) | 106 (20.9) |
| ≥4 | 1,275 (16.4) | 1,139 (16.5) | 136 (15.1) | 1,113 (16.2) | 50 (12.6) | 112 (22.1) |
| BMI category change (WHO) |  |  |  |  |  |  |
| Underweight-Underweight | 155 (2.0) | 135 (2.0) | 20 (2.2) | 136 (2.0) | 17 (4.3) | 10 (0.4) |
| Underweight-Normal weight | 209 (2.7) | 182 (2.6) | 27 (3.0) | 184 (2.7) | 15 (3.7) | 10 (2.0) |
| Underweight-Overweight | 9 (0.1) | 7 (0.1) | 2 (0.2) | 9 (0.1) | 0 (0.0) | 0 (0.0) |
| Underweight-Obesity | 3 (0.0) | 3 (0.0) | 0 (0.0) | 3 (0.0) | 0 (0.00) | 0 (0.0) |
| Normal weight-Underweight | 240 (3.1) | 203 (2.9) | 37 (4.1) | 222 (3.2) | 15 (3.8) | 3 (0.6) |
| Normal weight-Normal weight | 3,620 (46.5) | 3,185 (46.3) | 435 (48.3) | 3,255 (47.) | 203 (51.3) | 162 (32.0) |
| Normal weight-Overweight | 978 (12.5) | 879 (12.8) | 99 (11.0) | 859 (12.5) | 39 (9.8) | 80 (15.7) |
| Normal weight-Obesity | 169 (2.2) | 150 (2.2) | 19 (2.1) | 137 (2.0) | 9 (2.3) | 23 (4.5) |
| Overweight-Underweight | 9 (0.1) | 9 (0.1) | 0 (0.0) | 7 (0.1) | 0 (0.0) | 2 (0.4) |
| Overweight-Normal weight | 371 (4.8) | 312 (4.5) | 59 (6.5) | 332 (4.8) | 23 (5.8) | 16 (3.2) |
| Overweight-Overweight | 926 (11.9) | 832 (12.2) | 94 (10.4) | 826(12.0) | 31 (7.8) | 69 (13.8) |
| Overweight-Obesity | 389 (5.0) | 354 (5.2) | 35 (3.8) | 335 (4.9) | 9 (2.3) | 45 (9.0) |
| Obesity-Underweight | 3 (0.0) | 3 (0.0) | 0 (0.0) | 2 (0.0) | 0 (0.0) | 1 (0.2) |
| Obesity-Normal weight | 43 (0.6) | 40 (0.6) | 3 (0.3) | 37 (0.5) | 3 (0.8) | 3 (0.6) |
| Obesity-Overweight | 108 (1.4) | 92 (1.4) | 16 (1.8) | 91 (1.3) | 4 (1.0) | 13 (2.6) |
| Obesity-Obesity | 553 (7.1) | 496 (7.3) | 57 (6.3) | 447 (6.5) | 28 (7.1) | 78 (15.4) |
| % change weight between pregnancies |  |  |  |  |  |  |
| Lost weight | 2,231 (28.7) | 1,933 (28.1) | 298 (33.0) | 1,960 (28.5) | 148 (37.4) | 123 (24.3) |
| 0 to 8,62% | 2,777 (35.6) | 2,470 (35.9) | 307 (34.0) | 2,467 (35.8) | 137 (34.6) | 173 (34.1) |
| Gained >8,62% | 2,777 (35.7) | 2,479 (36.0) | 298 (33.0) | 2,455 (35.7) | 111 (28.0) | 211 (41.6) |

**Table S2**: Interpregnancy weight change and adverse births outcomes in the second pregnancy, Brazil, 2008-2015.

|  | | Preterm Birth (<37 gestational weeks) | | Low birth weight (<2500g) | | Macrosomia (≥4000g) | |
| --- | --- | --- | --- | --- | --- | --- | --- |
|  |  | Crude odds ratio  (95% CI) | Adjusted odds  ratio (95% CI)^a^ | Crude odds ratio  (95% CI)^a^ | Adjusted odds  ratio (95% CI)^a^ | Crude odds ratio  (95% CI) | Adjusted odds  ratio (95% CI)^a^ |
| BMI change (kg/m2) | | n= 7,785 | n= 7,222 | n= 7,785 | n= 7,222 | n= 7,785 | n= 7,222 |
| <−2 | | 1.32 (1.06-1.65) | 1.27 (1.01-1.60) | 1.08 (0.79-1.49) | 1.09 (0.78-1.52) | 0.99 (0.71-1.38) | 0.97 (0.68-1.37) |
| −2 to <−1 | | 1.32 (1.03-1.7) | 1.26 (0.96-1.64) | 1.11 (0.77-1.58) | 1.10 (0.72-1.60) | 0.96 (0.65-1.41) | 0.96 (0.64-1.42) |
| −1 to <1 | | 1.00 | 1.00 | 1.00 | 1.00 | 1.00 | 1.00 |
| 1 to <2 | | 0.99 (0.79-1.25) | 1.00 (0.78-1.26) | 0.71 (0.50-1.01) | 0.73 (0.51-1.06) | 1.23 (0.92-1.66) | 1.26 (0.93-1.71) |
| 2 to <4 | | 0.91 (0.74-1.13) | 0.96 (0.77-1.20) | 0.78 (0.58-1.06) | 0.85 (0.63-1.16) | 1.29 (0.99-168) | 1.28 (0.97-1.70) |
| ≥4 | | 0.94 (0.76-1.17) | 0.93 (0.74-1.18) | 0.69 (0.50-0.97) | 0.75 (0.53-1.06) | 1.61 (1.24-2.10) | 1.60 (1.21-2.12) |
| BMI category change (WHO) | | n= 7,718 | n= 7,158 | n= 7,718 | n= 7,158 | n= 7,718 | n= 7,158 |
| Underweight-Underweight | | 1.08 (0.67-1.75) | 1.20 (0.74-1.96) | 2.00 (1.19-3.38) | 2.15 (1.26-3.68) | 0.30 (0.07-1.20) | 0.30 (0.07-1.20) |
| Underweight-Normal weight | | 1.09 (0.72-1.65) | 1.10 (0.70-1.71) | 1.31 (0.76-2.25) | 1.49 (0.85-2.60) | 1.09 (0.57-2.10) | 0.96 (0.48-1.91) |
| Underweight-Overweight | | * | * | * | * | * | * |
| Underweight-Obesity | | * | * | * | * | * | * |
| Normal weight-Underweight | | 1.33 (0.93-1.92) | 1.43 (1.00-2.07) | 1.08 (0.63-1.86) | 1.09 (0.62-1.91) | 0.27 (0.09-0.86) | 0.19 (0.05-0.77) |
| Normal weight-Normal weight | | 1.00 | 1.00 | 1.00 | 1.00 | 1.00 | 1.00 |
| Normal weight-Overweight | | 0.82 (0.65-1.04) | 0.84 (0.66-1.07) | 0.73 (0.51-1.03) | 0.78 (0.55-1.30) | 1.90 (1.42-2.47) | 1.82 (1.36-2.44) |
| Normal weight-Obesity | | 0.93 (0.57-1.51) | 1.00 (0.62-1.70) | 1.05 (0.52-2.10) | 1.20 (0.60-2.41) | 3.38 (2.11-5.40) | 3.85 (2.39-6.21) |
| Overweight-Underweight | | * | * | * | * | * | * |
| Overweight-Normal weight | | 1.38 (1.03-1.86) | 1.28 (0.94-1.76) | 1.11 (0.71-1.73) | 1.09 (0.68-1.74) | 0.97 (0.57-1.64) | 0.81 (0.45-1.45) |
| Overweight-Overweight | | 0.83 (0.65-1.05) | 0.85 (0.66-1.09) | 0.60 (0.41-0.88) | 0.58 (0.38-0.87) | 1.68 (1.25-25) | 1.68 (1.24-2.28) |
| Overweight-Obesity | | 0.72 (0.5-1.04) | 0.72 (0.49-1.06) | 0.43 (0.22-0.85) | 0.47 (0.24-0.92) | 2.70 (1.90-3.82) | 2.61 (1.79-3.78) |
| Obesity-Underweight | | * | * | * | * | * | * |
| Obesity-Normal weight | | * | * | * | * | * | * |
| Obesity-Overweight | | 1.27 (0.74-2.19) | 1.38 (0.78-2.43) | 0.70 (0.26-1.94) | 0.60 (0.19-1.92) | 2.87 (1.57-5.24) | 3.12 (1.68-5.77) |
| Obesity-Obesity | | 0.84 (0.63-1.13) | 0.88 (0.65-1.18) | 1.00 (0.67-1.51) | 0.99 (0.65-1.50) | 3.51 (2.63-4.67) | 3.46 (2.55-4.69) |
| % change weight between pregnancies | | n= 7,785 | n= 7,222 | n= 7,785 | n= 7,222 | n= 7,785 | n= 7,222 |
| Lost weight |  | 1.24 (1.05-1.47) | 1.22 (1.02-1.46) | 1.36 (1.07-1.73) | 1.38 (1.08-1.78) | 0.89 (0.70-1.14) | 0.92 (0.72-1.18) |
| 0 to 8.62% |  | 1.00 | 1.00 | 1.00 | 1.00 | 1.00 | 1.00 |
| Gaine >8.62% |  | 0.97 (0.82-1.15) | 0.99 (0.83-1.18) | 0.81 (0.63-1.05) | 0.85 (0.65-1.12) | 1.23 (0.99-1.51) | 1.23 (0.99-1.54) |
| ^a^ adjusted analysis for residence area, maternal education, maternal age at delivery, all the first pregnancy, and maternal race and interval intersectional. N denotes the number of live births included in each analysis. Variations in the sample sizes are due to missing data in the covariates. *These categories were excluded from the analyses because of insufficient sample sizes. | | | | | | | |

**Table S3**: Interpregnancy weight change and preterm birth in the second pregnancy by pre-pregnancy BMI category in the 1^st^ pregnancy, Brazil, 2008-2015.

| % change  weight between pregnancies | BMI 1^st^ pregnancy | | | | | | | |
| --- | --- | --- | --- | --- | --- | --- | --- | --- |
|  | **Underweight (<18.5 Kg/m^2^)** | | **Normal weight**  **(18.5 to <25 Kg/m^2^ )** | | **Overweight (25 to <30 Kg/m^2^)** | | **Obesity (≥30 Kg/m^2^)** | |
|  | Crude odds  Rates (95% CI) | Adjusted odds  ratio (95% CI)^a^ | Crude odds rates (95% CI) | Adjusted odds  ratio (95% CI)^a^ | Crude odds  Rates (95% CI) | Adjusted odds  ratio (95% CI)^a^ | Crude odds rates (95% CI) | Adjusted odds  ratio (95% CI)^a^ |
| Lost weight | 1.83 (1.01-3.31) | 1.80 (0.98-3.33) | 1.18 (0.95-1.47) | 1.16 (0.92-1.46) | 1.45 (1.00-2.08) | 1.43 (0.97 – 2.11) | 1.03 (0.59-1.79) | 1.05 (0.59 – 1.87) |
| Gained 0 to 8,62 % | 1.00 | 1.00 | 1.00 | 1.00 | 1.00 | 1.00 | 1.00 | 1.00 |
| Gained ≥8,62% | 1.09 (0.68-1.77) | 1.03 (0.62-1.71) | 0.96 (0.78-1.19) | 0.98 (0.78-1.22) | 0.90 (0.60-1.37) | 0.95 (0.61-1.48) | 0.83 (0.44-1.60) | 1.02 (0.52-2.00) |
| ^a^ adjusted analysis for residence area, maternal education, maternal age at delivery, all the first pregnancy, and maternal race and interval intersectional. N denotes the number of live births included in each analysis. Variations in the sample sizes are due to missing data in the covariates. | | | | | | | | |

**Table S4**: Interpregnancy weight change and low birth weight in the second pregnancy by pre-pregnancy BMI category in the 1^st^ pregnancy, Brazil, 2008-2015.

| % change  weight between pregnancies | BMI 1^st^ pregnancy | | | | | | | |
| --- | --- | --- | --- | --- | --- | --- | --- | --- |
|  | **Underweight (<18.5 Kg/m^2^)** | | **Normal weight**  **(18.5 to <25 Kg/m^2^ )** | | **Overweight (25 to <30 Kg/m^2^)** | | **Obesity (≥30 Kg/m^2^)** | |
|  | Crude odds  Rates (95% CI) | Adjusted odds  ratio (95% CI)^a^ | Crude odds rates (95% CI) | Adjusted odds  ratio (95% CI)^a^ | Crude odds  Rates (95% CI) | Adjusted odds  ratio (95% CI)^a^ | Crude odds rates (95% CI) | Adjusted odds  ratio (95% CI)^a^ |
| Lost weight | 1.74 (0.86-3.50) | 1.67 (0.81-3.43) | 1.43 (1.05-1.94) | 1.44 (1.04-1.99) | 1.41 (0.77-2.59) | 1.64 (0.86-3.13) | 1.45 (0.66-3.19) | 1.49 (0.65-3.44) |
| Gained 0 to 8,62 % | 1.00 | 1.00 | 1.00 | 1.00 | 1.00 | 1.00 | 1.00 | 1.00 |
| Gained ≥8,62% | 0.88 (0.49-1.60) | 1.00 (0.54-1.84) | 0.75 (1.05-1.94) | 0.77 (0.54-1.09) | 0.83 (0.40-1.71) | 0.96 (0.44-2.08) | 1.11 (0.44-2.83) | 1.21 (0.45-3.25) |
| ^a^ adjusted analysis for residence area, maternal education, maternal age at delivery, all the first pregnancy, and maternal race and interval intersectional. N denotes the number of live births included in each analysis. Variations in the sample sizes are due to missing data in the covariates. | | | | | | | | |

**Table S5**: Interpregnancy weight change and macrosomia in the second pregnancy by pre-pregnancy BMI category in the 1^st^ pregnancy, Brazil, 2008-2015.

| % change  weight between pregnancies | BMI 1^st^ pregnancy | | | | | | | |
| --- | --- | --- | --- | --- | --- | --- | --- | --- |
|  | **Underweight (<18.5 Kg/m^2^)** | | **Normal weight**  **(18.5 to <25 Kg/m^2^ )** | | **Overweight (25 to <30 Kg/m^2^)** | | **Obesity (≥30 Kg/m^2^)** | |
|  | Crude odds  Rates (95% CI) | Adjusted odds  ratio (95% CI)^a^ | Crude odds rates (95% CI) | Adjusted odds  ratio (95% CI)^a^ | Crude odds  Rates (95% CI) | Adjusted odds  ratio (95% CI)^a^ | Crude odds rates (95% CI) | Adjusted odds  ratio (95% CI) ^a^ |
| Lost weight | 1.79 (0.42-7.63) | 1.94 (0.45-8.37) | 0.92 (0.65-1.30) | 0.91 (0.63-1.30) | 0.58 (0.36-0.92) | 0.64 (0.39-1.04) | 0.88 (0.53-1.48) | 0.96 (0.56-1.64) |
| 0 to 8,62 % | 1.00 |  | 1.00 | 1.00 | 1.00 | 1.00 | 1.00 | 1.00 |
| Gained ≥8,62% | 2.95 (1.07-8.17) | 2.55 (0.90-7.27) | 1.37(1.03-1.83) | 1.28 (0.95-1.73) | 1.09 (0.72-1.67) | 1.23 (0.79-1.93) | 1.14 (0.66-1.97) | 1.21 (0.67-2.18) |
| ^a^ adjusted analysis for residence area, maternal education, maternal age at delivery, all the first pregnancy, and maternal race and interval intersectional. N denotes the number of live births included in each analysis. Variations in the sample sizes are due to missing data in the covariates. | | | | | | | | |

| Table S6: Interpregnancy weight change and birth outcomes in the subsequent pregnancy by maternal age at in the 1^st^ pregnancy, Brazil, 2008-2015 | | | | | | | | | |
| --- | --- | --- | --- | --- | --- | --- | --- | --- | --- |
| Tirth  outcomes | **Adolescents (< 20 y o)** | | | | **Adults** | | | | |
|  | **Lost weight** | | **Gained >8.62%** | | **Lost weight** | | **Gained >8.62%** | | |
|  | Crude odds  Rates (95% CI) | Adjusted odds  ratio (95% CI) ^a^ | Crude odds  Rates (95% CI) | Adjusted odds  ratio (95% CI)^a^ | Crude odds  Rates (95% CI) | Adjusted  odds ratio (95% CI)^a^ | Crude odds  Rates (95% CI) |  | Adjusted odds  ratio (95% CI)^a^ |
| Preterm | 1.20 (9.1-1.59) | 1.12 (0.84-1.05) | 0.93 (0.72-1.21) | 0.97 (0.74-1.27) | 1.26 (1.02-1.22) | 1.29 (1.03-1.61) | 0.97 (0.78-1.22) |  | 1.01 (0.80-1.28) |
| Low birth weight | 1.78 (1.20-2.65) | 1.85 (1.22-2.85) | 0.96 (0.64-1.45) | 1.01 (0.65-1.58) | 1.17 (0.86-1.56) | 1.16 (0.86-1.58) | 0.73 (0.53-1.03) |  | 0.77 (0.55-1.10) |
| Macrosomia | 0.81 (0.51-1.29) | 0.87 (0.54-1.41) | 1.17 (0.80-1.70) | 1.20 (0.80-1.80) | 0.92 (0.70-1.22) | 0.93 (0.70-1.25) | 1.31 (1.02-1.68) |  | 1.24 (0.95-1.62) |
| ^a^ adjusted analysis for residence area, maternal education, maternal age at delivery, all the first pregnancy, and maternal race and interval intersectional. N denotes the number of live births included in each analysis. | | | | | | | | | |
